# Autism-Like Behavior and Epigenetic Changes Associated with Autism as Consequences of *In Utero* Exposure to Environmental Pollutants in a Mouse Model

**DOI:** 10.1155/2015/426263

**Published:** 2015-10-26

**Authors:** Denise S. Hill, Robert Cabrera, Deeann Wallis Schultz, Huiping Zhu, Wei Lu, Richard H. Finnell, Bogdan J. Wlodarczyk

**Affiliations:** ^1^Center for Environmental and Genetic Medicine, Institute of Biosciences and Technology, Texas A&M University System Health Science Center, Houston, TX 77030, USA; ^2^Department of Nutritional Sciences, The University of Texas at Austin, Austin, TX 78723, USA; ^3^Department of Biochemistry & Biophysics, Texas A&M University, College Station, TX 77843, USA

## Abstract

We tested the hypothesis that *in utero* exposure to heavy metals increases autism-like behavioral phenotypes in adult animals and induces epigenetic changes in genes that have roles in the etiology of autism. Mouse dams were treated with cadmium, lead, arsenate, manganese, and mercury via drinking water from gestational days (E) 1–10. Valproic acid (VPA) injected intraperitoneally once on (E) 8.5 served as a positive control. Young male offspring were tested for behavioral deficits using four standardized behavioral assays. In this study, *in utero* exposure to heavy metals resulted in multiple behavioral abnormalities that persisted into adulthood. VPA and manganese induced changes in perseverative/impulsive behavior and social dominance behavior, arsenic caused changes only in perseverative/impulsive behavior, and lead induced abnormalities in social interaction in comparison to the control animals. Brain samples from Mn, Pb, and VPA treated and control animals were evaluated for changes in CpG island methylation in promoter regions and associated changes in gene expression. The Chd7 gene, essential for neural crest cell migration and patterning, was found to be hypomethylated in each experimental animal tested compared to water-treated controls. Furthermore, distinct patterns of CpG island methylation yielded novel candidate genes for further investigation.

## 1. Introduction

Autism spectrum disorder (ASD), commonly referred to as autism, is diagnosed in 1 of every 68 children in the United States and is characterized by a range of complex neurodevelopment disorders. Diagnostic and Statistical Manual of Mental Disorders (DSM-5) define ASD as a neurobehavioral disorder manifested by persistent deficits in social and communication interaction, deficits in developing, understanding, and maintaining relationships, and abnormal and fixed interests and repetitive behavior [1]. Accumulating evidence suggests that epigenetic factors play a strong role in the etiology of ASD [2–6]. Epigenetic disruption of regulatory CpG islands has also been associated with autism. For example, a decrease in expression of the methyl binding protein gene,* MECP2*, was observed in the brain tissue of autistic patients. Downregulation of MeCP2 was also demonstrated in valproate animal model of autism [7]. This gene functions as a transcriptional repressor at specific gene promoters crucial to brain development [8, 9].

Valproic acid (VPA) is a commonly used antiepileptic drug.* In utero* exposure to VPA increases the risk for neural tube defects (NTDs) in humans [10, 11] and laboratory rodents [12]. VPA is also known to cause behaviors consistent with some aspects of autism and is employed as an established research model for inducing autism [13, 14]. Years of preliminary data suggest that there is a genetic basis underlying VPA-sensitivity to the induction of neural deficits in laboratory animals [15–17]. VPA is also known to affect the epigenome, as well as blood levels of folate and homocysteine, both of which have the potential to dysregulate critical developmental genes that can result in adverse pregnancy outcomes [18, 19]. A growing body of literature indicates that similar disruption is observed in some autistic patients: both disrupted folate and homocysteine status [20–22] and epigenomic disruption of regulatory CpG islands have been associated with autism [23]. Furthermore, evidence suggests that exposure to environmental neurotoxins is a risk factor for autism [24].

Industrial chemicals are being produced and released into the environment at a staggering rate. The average consumer is exposed to up to 10,000 different chemicals every day out of a world market that includes over 30,000 industrial chemicals sold at quantities greater than 400 million tons per year [25]. These chemicals range in toxicity from being benign to being extremely hazardous and the environmental and public health implications of this overwhelming and pervasive exposure have not been well characterized for the vast majority of chemicals. Among the hazardous toxicants, heavy metals, known neurotoxicants continue to be of a great concern due to their increasing anthropogenic presence in the environment [26]. Several epidemiological studies reported a relationship between autism and heavy metal biomarkers [27, 28]. Exposure to industrial chemicals has been thought to play a role in the etiology of autism through epigenetic mechanisms. This is based on evidence suggesting the plausibility of a role for mercury in the etiology of autism [29, 30], and mercury in maternal peripheral and cord blood has been shown to affect fetal epigenetic status [31]. A handful of reports also points out to other heavy metals neurotoxicity and epigenetic mechanism [32, 33].

The experiments presented in this paper were designed to identify abnormal neurobehavioral phenotypes resulting from* in utero* exposure to several identified environmental pollutants. We hypothesized that* in utero* exposure to these toxicants results in epigenetic changes in key developmental genes, affecting pathways that have direct roles in the etiology of autism. Collectively, this work provides an assessment of the effect of* in utero* exposure to selected heavy metals on the epigenome and associated autistic-like phenotypes in the offspring that persist as adult behaviors.

## 2. Materials and Methods

### 2.1. General Study Design

C57BL/6J mice were exposed* in utero* to selected toxicants and evaluated for their adult behavioral phenotype. VPA was used as a positive control because it is a widely used mitochondriotoxic medication that induces a spectrum of teratogenic effects including behavioral disruption and is used as a rodent model for studying autistic behavioral changes [34]. Environmental toxicants including cadmium (Cd), lead (Pb), arsenic (As), manganese (Mn), and mercury (Hg) were investigated with normal drinking water used as a negative control.

### 2.2. Animals and Housing

C57B6/J mice were housed in the Institute of Biosciences and Technology Vivarium, which is fully accredited by the Association for Assessment and Accreditation of Laboratory Animal Care. The animals were maintained in clear polycarbonate microisolator cages and were allowed free access to food and water (Harlan Teklad Rodent Diet #8606, Ralston Purina, St. Louis, MO). The mice were maintained on a 12 h light/dark cycle. Nulligravid females, 50–70 days of age, were mated overnight with males and examined for the presence of vaginal plugs the following morning and the onset of gestation was considered to be 10 p.m. of the previous night, the midpoint of the dark cycle.

### 2.3. Treatment with Select Compounds

Presumed gravid dams C57BL/6J were treated with one of seven agents: drinking water served as the negative control and VPA (CAS# 1069-66-5, valproic acid sodium salt, ≥98%, Sigma-Aldrich Chemicals, St. Louis, MO) administered at 600 mg/kg by intraperitoneal injection on gestational day 8.5 only served as the positive control. Five groups were treated from the first day of pregnancy through day 10.5, the end of neurulation, which generally occurs from E8.0–10.5, via treated drinking water containing 10 ppm Cd as cadmium chloride (CAS# 10108-64-2, 99.99% trace metals basis, Sigma-Aldrich Chemicals, St. Louis, MO), 300 ppm Pb as lead acetate (CAS# 6080-56-4, lead(II) acetate trihydrate, 99.999% trace metals basis, Sigma-Aldrich Chemicals, St. Louis, MO), 0.5 ppm As as sodium arsenate (CAS# 10048-95-0, sodium arsenate heptahydrate, ACS Reagent, ≥98%, Sigma-Aldrich Chemicals, St. Louis, MO), 10 ppm Mn as manganese chloride (CAS# 13446-34-9, manganese(II) chloride tetrahydrate, 99.99% trace metals basis, Sigma-Aldrich Chemicals, St. Louis, MO), or 20 ppm Hg as mercury chloride (CAS# 7487-94-7, mercury(II) chloride, ACS reagent, ≥99.9%, Sigma-Aldrich Chemicals, St. Louis, MO). Treated dams maintained in separate cages were allowed to give birth. Only animals from litters consisting of 6–8 pups were admitted for further testing. All experimental mice were weaned at three weeks of age and maintained in separate cages. Because of the higher incidence of autism reported in male children as compared to female children, male mice were used exclusively in these studies. Twelve mice per group that is seventy two males in total were used in this study. Animals were assigned randomly to experimental groups and to reduce any litter effect only one male from a given litter was assigned to the same treatment group (no littermates in the same group).

### 2.4. Behavioral Assays and Statistical Analysis

The behavioral testing began when experimental animals reached twelve weeks of age and lasted until twenty weeks of age with at least one-week break period between consecutive assays for any individual mouse. All treatment groups were evaluated with 4 different neurobehavioral assays: nest building to begin to assess behavioral deficits; marble burying for perseverative/impulsive behaviors; 3-chambered box for social interaction; and tube test for social dominance. Each assay was performed sequentially, such that the least stressful assay was performed first and the most stressful performed last: nest building, marble burying, 3-chambered box for social interaction, and tube test.

#### 2.4.1. Nest Building

Upon reaching twelve weeks of age, mice were tested for their ability to build nests. C57 mice are considered exceptionally robust nest builders, a normal behavior for both male and female mice. Nesting has been reported to be impaired by brain lesions including those to the hippocampus and medial prefrontal cortex [35], pharmaceutical intervention with selective serotonin reuptake inhibitors (SSRIs) [36], and disease (e.g., scrapie) [37]. Nestlets (Ancare, Bellmore, NY), manufactured from pulped virgin cotton fiber and sterilized during the manufacturing process, were routinely provided in each cage. Three grams of Nestlet was placed in each cleaned cage 1 hour prior to the onset of the dark cycle and left undisturbed overnight. Nest building was assessed based on a 5-point scale, 1: Nestlet not noticeably touched; 2: Nestlet partially torn; 3: Nestlet mostly shredded, but no identifiable nest; 4: identifiable, but flat nest; and 5: a near perfect nest. Data were analyzed using a nonparametric ANOVA (Kruskal-Wallis test) with Dunn's multiple comparison test.

#### 2.4.2. Marble Burying

Mice were tested for perseverative/impulsive behavior using marble burying as an index. As mice dig, a normal behavior, marbles are incidentally submerged in litter. It is suggested that this is a genetically regulated behavior which is not related to anxiety or novelty [38, 39]. This repetitive/perseverative behavior has been observed to be disrupted by a wide array of agents that include hippocampal lesions [40], NK1 antagonists [41], and many more [42–44]. Mice were presented with 16 marbles in a 4 × 4 grid placed lightly on a gently tamped volume of aspen chip bedding 5 cm deep and allowed 30 minutes to bury marbles. Marbles were considered buried if >70% of the marble volume was submerged. The numbers of marbles buried were recorded and analyzed using a nonparametric ANOVA (Kruskal-Wallis test) with Dunn's multiple comparison test.

#### 2.4.3. Three-Chambered Social Testing Apparatus

In this assay we tested the normal preference of a mouse to investigate and spend time with a novel strange mouse, as opposed to a novel object. The three-chambered social testing apparatus was designed at NIH in order to measure various aspects of sociability in mice [45]. Three chambers are linked by closeable gates; transitions between chambers and time spent in each chamber may be recorded by a computerized monitoring program.

The test mouse was placed in the closed central chamber (#2) of the apparatus and allowed to acclimate for ten minutes. Next, a novel strange mouse was placed under an inverted wire cup in chambers #1 or #3, with a duplicate cup placed in the remaining chamber, the gates between the chambers were raised, and the behavior of the mouse was recorded for ten minutes. Total time spent with the novel strange mouse versus the novel object was analyzed with a 2-tailed paired *t*-test for each treatment group. Failure to achieve a statistically significant preference for spending time with a novel stranger was considered indicative of abnormal behavior. A set of novel stranger mice had been previously acclimated to being restrained for 10-minute periods within the confines of the inverted cup. The stranger mice were alternated from side one to side three to ensure that a preference for a specific side by the test mice did not confound the outcome. Similarly, the apparatus was housed in a room with an ambient noise level of 55 db, provided by the ventilation fan and placed directly under a light. The apparatus was wiped down with 70% ethanol at the onset of investigations and between mice thereafter.

#### 2.4.4. Tube Test for Social Dominance

Mice were tested for a preference, when challenged with a novel strange mouse at opposing ends of a 12′′ long PVC pipe, to yield to the novel stranger and back out of the tube (lose) or complete transit of the tube while the novel stranger yields (win). Disruption of normal behavior has been reported in mice that recapitulate many characteristic features of autism-like behavior, such as those with a truncated MeCP2 that recapitulate many features of Rett Syndrome [46] and chromosome deletions causing classic features of Williams-Beuren syndrome, such as hypersociability [47]. Each test mouse was challenged three times with a novel strange mouse for a maximum allotted time of four minutes (a match). Mice were placed headfirst at opposite ends of a tube and released simultaneously. The match ended when one retreated from the tube and was assigned a score of zero (lose); the remaining mouse was assigned a score of one (win). Stranger mice were generally used three times and no more than four times. Both test mice and stranger mice were separately acclimated to the testing apparatus in advance of testing. Failure to achieve a win/loss outcome in four minutes, or the mice crossing over each other to exit opposite ends of the tube, excluded the match from scoring. The outcome from the three matches for each mouse was calculated as an average percentage of wins and then the mean percentage of win matches was calculated for each treatment group. These mean values were analyzed using a nonparametric ANOVA (Kruskal-Wallis test) with Dunn's multiple comparison test.

### 2.5. Tissue Harvest and Molecular Assays

Three animals from negative control group and from each of the groups that exhibited changes in the greatest number of behavioral assays (water control, VPA, Mn, and Pb) were evaluated for changes in frontal cortex CpG island methylation in promoter regions and associated changes in gene expression that similarly persisted into adulthood.

After all behavioral assays were complete, experimental mice were euthanized via CO_2_ asphyxiation and brain tissue immediately harvested. The frontal cortex was dissected out and divided for extraction of DNA and RNA used in subsequent molecular assays. Agilent's Mouse CpG Island Microarray and Genomic DNA Enzymatic Labeling Kit (Agilent, Santa Clara, CA) were used to identify changes in methylation of genes previously known to be important for early neurodevelopment, as well as identifying novel candidate genes. The 2 × 105K format interrogates over 16,000 CpG islands using 97,652 probes in or within 95 bp of CpG islands and was sourced from UCSC mm8 (NCBI Build 36).

Briefly, genomic DNA was isolated and subsequent performance of the array was performed by MOgene (St. Louis, MO). Whole genomic DNA was sheared (reference DNA) and labeled with Cy5 (blue). Methylated DNA was precipitated from each sample and labeled with Cy3 (red). The samples were then hybridized to the slide for 40 hours followed by washing, drying, and imaging. Data were extracted using the Feature Extraction software and methylation protocol. In order to view and search for high-level patterns in the results for each experimental analysis, hierarchical clustering was performed. Data tables were prepared as tab-delimited text files and loaded into Hierarchical Clustering Explorer 3.0 (University of Maryland, College Park, MD).

Upon completion of data analysis, microarray results were validated by real-time quantitative PCR using TaqMan Gene Expression Assays on an ABI 3730 DNA 7900HT Genetic Analyzer (Life Technologies, Carlsbad, CA) for those genes identified as having altered histone methylation. Gene-specific probes and primer sets were purchased from Life Technologies. 50 ng cDNA of each sample was mixed with 10 *μ*L TaqMan Gene Expression PCR Master Mix and 1 *μ*L probe and primer mixture in a total volume of 20 *μ*L in a 384-well plate. The assays were performed according to the manufacturer's protocol on an ABI PRISM 7900HT Sequence Detection System. The thermocycling started with a 10 min denaturing at 95°C, followed by 40 cycles of 95°C for 15 sec and 60°C for 1 min. Data was analyzed using SDS software v2.1. Mouse* Actb* gene was used as internal control. ΔΔCt method was used to generate relative quantitative values (fold change). Each reaction was replicated two times and the mean value was used in final comparisons.

## 3. Results

### 3.1. Behavioral Assays

#### 3.1.1. Nest Building Assay

All mice in this study made nests that scored a 5 on a whole integer scale of 1–5. No statistically significant differences between the nests of water-treated control mice and those of mice exposed to toxicants* in utero* were observed.

#### 3.1.2. Marble-Burying Test

**Figure 1:**
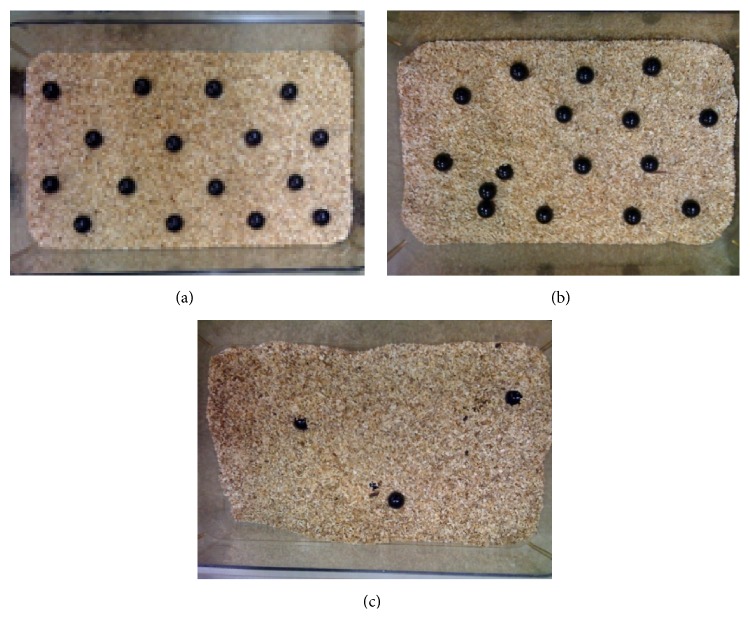
Marble-burying apparatus. (a) Marble-burying apparatus ready for use. (b) Marble-burying apparatus after use by representative VPA-treated animal. (c) Marble-burying apparatus after use by representative normal control animal.

**Figure 2:**
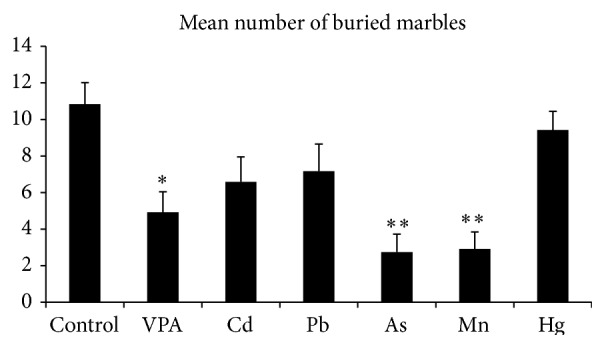
Marble-burying test for perseverative/repetitive behavior: VPA, As, and Mn exposed animals bury significantly fewer marbles than controls. Data were analyzed using the nonparametric ANOVA (Kruskal-Wallis test) with Dunn's multiple comparison test. *∗* denotes statistical significance (*p* < 0.05); *∗∗* denotes statistical significance (*p* < 0.001).

C57 mice were vigorous diggers, with water-treated controls burying nearly 70% of the marbles placed on the surface of the lightly packed bedding. Animals from all treated groups buried fewer marbles but statistically significant difference was confirmed only in three groups. VPA, As, and Mn exposed animals buried significantly fewer marbles than water-treated control mice with the *p* values less than 0.05, 0.01, and 0.01, respectively (Figures 1 and 2).

#### 3.1.3. Three-Chambered Testing Apparatus for Social Interaction

**Figure 3:**
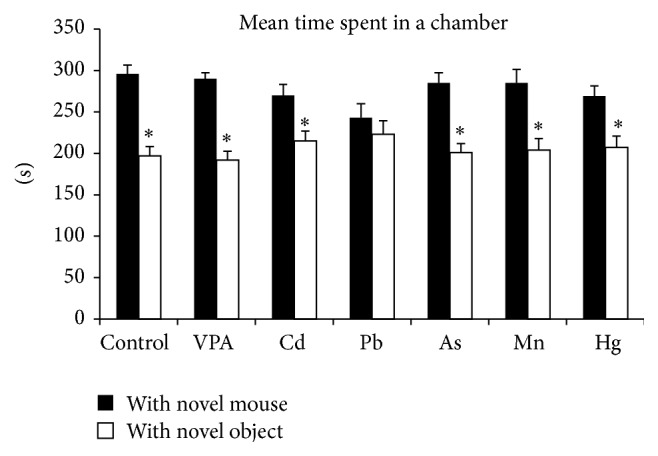
Three-chambered testing apparatus for social interaction: mice from all groups except the Pb exposed group exhibited a normal preference to spend significantly more time with a novel stranger mouse rather than with a novel object. Data were analyzed with a 2-tailed paired *t*-test for each treatment group. The preference was statistically significant in all groups (0.002 < *p* < 0.04 range), but Pb treated group (*p* > 0.05). *∗* denotes statistical significance.

Exposure to one toxicant, Pb, resulted in an abnormal lack of preference for spending time with a novel stranger mouse. Statistical analysis revealed that mice treated with Pb did not spend significantly more time with novel stranger mouse than with the novel object (*p* = 0.396). Water-treated control mice, as well as all other treatment groups, with the exception of Pb, displayed a normal preference for spending time with a novel stranger mouse rather than with a novel object. The observed differences (time with strange mouse − time with a novel object) were statistically significant with a *p* values ranging from 0.003 to 0.0001 (Figure 3).

#### 3.1.4. Tube Test for Social Dominance

Mice from water-treated control group on average won half (49.5%) of their matches. On the other hand, the VPA-exposed mice experienced significantly more losses (only 22% wins) as compared to water-treated controls (*p* < 0.05). The Mn-exposed mice experienced even more losses (only 19.2% wins *p* < 0.01). The tube test identified also abnormal behavior of th Pb-exposed mice. Even though the Pb-exposed group won 44.3% of their matches (not significantly different form control group *p* > 0.05), these matches were very long and 25% of the matches were unscored when the animals crossed over one another to exit the tube or simply remained in the tube together for the full 4 minutes of the match period. This behaviour was not observed in any other tested group (Table 1).

#### 3.1.5. Overview of Behavioral Studies

While none of the treatment groups displayed abnormal behavior in all four assays, 3 treatment groups displayed abnormal behavior in 2 of the assays when compared to water-treated controls: VPA (marble burying and tube test), Mn (marble burying and tube test), and Pb (three-chambered testing apparatus for social interaction and tube test). Molecular assays were performed using tissue from the frontal cortex of representative animals: three animals from each of the most affected groups (VPA, Mn, and Pb) and water control were used (Table 2).

### 3.2. Molecular Assays

#### 3.2.1. Mouse CpG Island Microarray for Altered Histone Methylation

**Figure 4:**
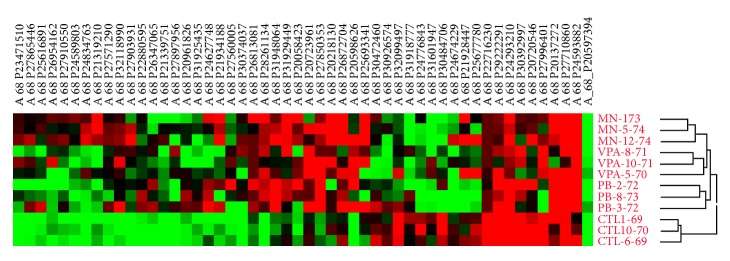
Mouse CpG Island Microarray for altered histone methylation. Hierarchical clustering of the untransformed data was done using Average Linkage (UPGMA) with similarity represented by Euclidean Distance. Data filtering: the genomic results were filtered from the UCSC (http://genome.ucsc.edu/) Mouse Genome Browser Gateway (NCBI37/mm9) for being within a CpG island and 5′ of a known gene. The samples clustered according to the treatment type.

CpG array probe names were associated with gene IDs (UCSC Genome Browser) and analyzed for significant differences using Student's *t*-test (*p* < 0.05) and a twofold change in average probe signal for treated versus control groups (Table 3). Clustering on the untransformed data using Average Linkage (UPGMA) with similarity represented by Euclidean Distance indicated that clusters fit expected groups based on treatment type (Figure 4). Further, one region was found with lower regulation in all treatment groups. This region is 5′ of the chromodomain-helicase-DNA-binding protein 7 (*Chd7*) gene, which was found to be hypomethylated in each sample tested from the three experimental groups displaying the most altered behavior (Table 3).

#### 3.2.2. Enrichment Analysis

A web-based analysis toolkit (WEB-based Gene SeT AnaLysis Toolkit, WebGestalt) [48] was utilized for enrichment analysis of genes adjacent to CpG enriched regions from the CpG microarray. Specifically,* M. musculus* was queried using gene symbols. Enrichment analysis utilized genome query, hypergeometric method, with significance set at *p* < 0.05 with Bonferroni correction. Gene enrichment is reported for Gene Ontology. For VPA, anatomical structure formation involved in morphogenesis (GO:0048646) was the most significantly (*p* < 0.005) enriched biological process. The genes in this group (Rab3gap1, Ptprz1, Col2a1, Chd7, and Nf2) also populated subsets specific to face development (GO:0060324) and camera-type eye development (GO:0043010). The synapse (GO:0045202) was the most significantly (*p* < 0.005) enriched cellular component in VPA treated animals. Collectively, altered facial and neurological development and dysregulated cellular signaling were indicated. The most significantly enriched GO groups with Mn exposure included cellular biosynthetic process (*p* < 0.01) under the biological process grouping (GO:0044249), transferase (GO:0016740) activity under molecular function (*p* < 0.01), and intracellular membrane-bounded organelle (GO:0043231) under cellular component (*p* < 0.05). These data are consistent with an overall impact on biosynthesis, with specific interaction of calmodulin and transferase activity. In animals exposed to lead* in utero*, the organelle (GO:0043226) cellular component group (*p* < 0.05) and protein kinase inhibitor activity (GO:0004860) molecular function group (*p* < 0.001) were the most significantly enriched.

Enrichment analysis was also conducted for animal phenotypes. Specifically, these gene groups produce a common phenotype when knocked out or mutated. The most significantly enriched gene groups by phenotypes were abnormal CNS glial cell morphology (Ptprz1, Pi4k2a, and App; MP:0000952) and head bobbing (Col2a1, Chd7; MP:0001410) for VPA treatment (*p* < 0.05), decreased body weight (Ovol1, Trib2, Slc13a5, Lrp5, Hk2, Pi4k2a, Chd7, and Tmeff2; MP:0001262) for Mn (*p* < 0.05), and impaired righting response (Trib2, Dst, Chd7, and Bsn; MP:0001523) for Pb (*p* < 0.001). Pb had an additional twelve significantly enriched phenotype groups that included premature death (MP:0002083), abnormal involuntary movement (MP:0003492), gliosis (MP:0002183), and abnormal cochlear nerve compound action potential (MP:0004415).

#### 3.2.3. Real-Time Q-PCR Validation

*Chd7* was overexpressed in all three (Mn, Pb, and VPA) treatment groups, as predicted by microarray data for altered histone methylation. When compared to water control group the expression of* Chd7* was 2.4-, 4.7-, and 1.6-fold higher in Mn, Pb, and VPA treated groups, respectively. While loss of function has been demonstrated to be responsible for CHARGE syndrome, in which affected children may have learning disabilities and craniofacial malformations, it is unknown at this time what effects overexpression due to hypomethylation might confer.

## 4. Discussion

C57 male mice were exposed* in utero* from the onset of gestation and during neural tube closure to toxicants suspected of affecting neurodevelopment in humans: VPA (the positive control treatment), lead, arsenic, cadmium, manganese, and mercury. Abnormal behavior was observed, compared to water-treated controls, in several treatment groups: VPA; manganese and lead. The observed impacts of VPA treatment were consistent with those found in the literature [34, 49, 50]. These data also indicate that prenatal manganese and lead exposures are associated with adverse neurobehavioral development in the resultant offspring.

In recent years, growing evidence suggests that both genetic and environmental factors contribute to ASDs. There are indications that certain gene variants confer genetic susceptibility [51]. Additionally,* de novo* mutations and advanced parental age have also been identified as genetically related risk factors [52, 53]. The observed pathophysiology of ASD in the central nervous system, including oxidative stress, neuroinflammation, and mitochondrial dysfunction, is consistent with environmental exposures to air pollution, organophosphates, and heavy metals [54].

It has been reported that exposure to industrial chemicals during early fetal development can cause brain injury at doses much lower than that affecting adult brain function [55]. The research on heavy metal exposure and ASD has largely been focusing on mercury; however, association between an ASD diagnosis and other toxins including lead and manganese has been observed in epidemiology studies [56, 57].

The knowledge on acute neurotoxic effect of lead can be traced back to the Roman times [58]. During the 1970s, wide spread subclinical neurobehavioral deficits such as problems with concentration, memory, and cognition were observed among children with raised blood lead levels [59–64]. More recent studies suggest that even low lead exposure could have greater than previously expected adverse effects on human brain development [65]. Exposure to low doses of manganese in children who are particularly susceptible may lead to subclinical neurotoxicity [55]. Recent studies have reported that manganese levels and psychomotor and mental development in children are significantly associated and the relationship presents as an inverted U-shape [66–68]. Despite the profound knowledge regarding neurotoxicity of industrial chemicals, the relationship between prenatal exposure to low doses of neurotoxic metals and neurodevelopment in the offspring remains unclear.

One recent epidemiology study [69] in a Chinese population revealed that high levels of manganese and lead in umbilical cord blood are associated with neurodevelopmental deficit at two years of age. Further, there may be an interactive effect between manganese and lead that aggravate this outcome. We used rodent models to carefully evaluate the spectrum of neurodevelopmental effects caused by common heavy metals. Our results are consistent with Lin and coworkers observation in humans and provided supporting evidence to fill the data gap on* in utero* exposure to selected neurotoxins.

When frontal cortex tissue from representative animals from treatment groups and water-treated controls was evaluated for changes in CpG island methylation, data clustering indicated that changes in methylation showed clear patterns: clusters fit expected groups based on treatment type. Further, one region was found with lower regulation in all treatment groups. This region is 5′ of the chromodomain-helicase-DNA-binding protein 7 (Chd7) gene. Real-time qPCR confirmed that* Chd7* was overexpressed by several fold changes in all three treatment groups, as predicted by microarray data.

While loss of function of this gene has been demonstrated to be responsible for CHARGE syndrome, in which affected children often have learning disabilities and craniofacial malformations, it is unknown at this time what effects overexpression due to hypomethylation might confer.

In this study,* in utero* exposure to several toxicants early in development resulted in behavioral abnormalities and changes in methylation of CpG islands and associated changes in expression that were evident in adulthood. The strength of the study is the combination of four behavioral assays, as well as molecular assays on gene expression and methylation status of genes responsive to exposure of toxicants. Investigation of the role of* Chd7* and other genes whose altered methylation state is associated with disrupted social behavior is warranted. Further, epidemiology studies suggested potential convergent interactions among neurotoxins and this hypothesis should be examined in rodent models as described in this study.

## Figures and Tables

**Table 1:** Tube test for social dominance.

Treatment	% wins	*p* value
Ctl (*n* = 36)	49.5	
VPA (*n* = 36)	22.0	<0.05
Cd (*n* = 36)	30.3	>0.05
Pb (*n* = 36)	44.3^a^	>0.05
As (*n* = 36)	52.5	>0.05
Mn (*n* = 36)	19.2	<0.01
Hg (*n* = 36)	28.9	>0.05

The mean values were analyzed using a nonparametric ANOVA (Kruskal-Wallis test) with Dunn's multiple comparison test.

^a^25% of matches crossed over or “timed out,” leaving the match unscored.

**Table 2:** Metatable for behavioral studies.

Treatment	Marble burying	3-chambered box	Tube test
VPA (*n* = 12)	X		X
Cd (*n* = 12)			
Pb (*n* = 12)		X	#
As (*n* = 12)	X		
Mn (*n* = 12)	X		X
Hg (*n* = 12)			

Groups that displayed behaviors significantly different from expected outcome are marked with X.

# indicates that significant numbers of test animals had an unscored match.

**Table 3:** Differentially methylated regions and associated genes.

VPA			Manganese			Lead		
Gene ID	CpG	VPA : Ctl	Gene ID	CpG	Mn : Ctl	Gene ID	CpG	Pb : Ctl
*Col2a1*	chr15	−1.25942	*Atf1*	chr15	−2.40606	*Lpo*	chr11	−2.1686
*Glt25d2*	chr1	−1.21282	*Lrp5*	chr19	−1.53512	*Alox5*	chr6	−1.94012
*App*	chr16	−1.15531	*Ercc3*	chr18	−1.42284	*Arhgef10*	chr8	−1.61243
***Chd7***	**chr4**	−1.11644	*Pnpla3*	chr15	−1.32399	*Pkia*	chr3	−1.29882
*Ptprz1*	chr6	−1.0547	*Gpr120*	chr19	−1.18607	*Frmd4b*	chr6	−1.28576
*Nf2*	chr11	1.022569	*Rasgrf2*	chr13	−1.13359	***Chd7***	**chr4**	−1.14022
*Rab3gap1*	chr1	1.059968	***Chd7***	**chr4**	−1.06245	*Derl2*	chr11	1.021783
*Nmnat2*	chr1	1.134621	*Hivep2*	chr10	1.032248	*Ctsf*	chr19	1.023935
*Eif5a*	chr11	1.442196	*Ctxn1*	chr8	1.063293	*Bsn*	chr9	1.028517
*Pi4k2a*	chr19	1.548454	*Ovol1*	chr19	1.087982	*Odz2*	chr11	1.066651
*R3hdm1*	chr1	2.132963	*Hk2*	chr6	1.132845	*Rpp38*	chr2	1.205103
			*Slc13a5*	chr11	1.200217	*Mtx2*	chr2	1.403068
			*Adcy1*	chr11	1.272176	*Fads3*	chr19	1.511005
			*Scn5a*	chr9	1.381722	*Plxna1*	chr6	1.563187
			*Hs3st3a1*	chr11	1.454418	*Zbtb5*	chr4	1.564412
			*Pi4k2a*	chr19	1.532898	*Dguok*	chr6	1.565438
			*Dmtf1*	chr5	1.597027	*Dnm2*	chr9	1.584182
			*Gdap1*	chr1	1.676317	*Trib2*	chr12	1.654498
			*Csmd1*	chr8	1.730636	*Prpsap2*	chr11	1.692807
			*Tmeff2*	chr1	1.771945	*Dst*	chr1	2.000012
			*Trib2*	chr12	1.790036			
			*Hat1*	chr2	1.840714			
			*Prmt8*	chr6	1.858983			
			*Mrps28*	chr3	1.916268			
			*Tnrc6b*	chr15	1.969439			

CpG array probe names were associated with gene IDs (UCSC Genome Browser). All array data was normalized to the internal sheered standard and control data was compared to experimental data for differential methylation using log_2_⁡ ratios. Reported significant differences were defined by >2-fold change, *t*-test (*p* < 0.005). The resulting changes in methylation for exposures were 11 for VPA (5 down, 6 up), 25 for Mn (7 down, 18 up), and 20 for Pb (6 down, 14 up).
